# Menstrual Cycle Phase Modulates Auditory-Motor Integration for Vocal Pitch Regulation

**DOI:** 10.3389/fnins.2016.00600

**Published:** 2016-12-27

**Authors:** Xiaoxia Zhu, Yang Niu, Weifeng Li, Zhou Zhang, Peng Liu, Xi Chen, Hanjun Liu

**Affiliations:** ^1^Department of Rehabilitation Medicine, The Sixth Affiliated Hospital of Sun Yat-sen UniversityGuangzhou, China; ^2^Department of Rehabilitation Medicine, Anhui No. 2 Province People's HospitalHefei, China; ^3^Department of Rehabilitation Medicine, The First Affiliated Hospital of Sun Yat-sen UniversityGuangzhou, China; ^4^Guangdong Provincial Key Laboratory of Brain Function and Disease, Zhongshan School of Medicine, Sun Yat-sen UniversityGuangzhou, China

**Keywords:** auditory feedback, auditory-vocal integration, menstrual cycle, estradiol, progesterone

## Abstract

In adult females, previous work has demonstrated that changes in auditory function and vocal motor behaviors may accompany changes in gonadal steroids. Less is known, however, about the influence of gonadal steroids on auditory-motor integration for voice control in humans. The present event-related potential (ERP) study sought to examine the interaction between gonadal steroids and auditory feedback-based vocal pitch regulation across the menstrual cycle. Participants produced sustained vowels while hearing their voice unexpectedly pitch-shifted during the menstrual, follicular, and luteal phases of the menstrual cycle. Measurement of vocal and cortical responses to pitch feedback perturbations and assessment of estradiol and progesterone levels were performed in all three phases. The behavioral results showed that the menstrual phase (when estradiol levels are low) as associated with larger magnitudes of vocal responses than the follicular and luteal phases (when estradiol levels are high). Furthermore, there was a significant negative correlation between the magnitudes of vocal responses and estradiol levels. At the cortical level, ERP P2 responses were smaller during the luteal phase (when progesterone levels were high) than the menstrual and follicular phases (when progesterone levels were low). These findings show neurobehavioral evidence for the modulation of auditory-motor integration for vocal pitch regulation across the menstrual cycle, and provide important insights into the neural mechanisms and functional outcomes of gonadal steroids' influence on speech motor control in adult women.

## Introduction

Gonadal steroids, such as estradiol and progesterone, have demonstrable effects on behavior and neuronal activity involved in cognitive functions, emotional control, and sensory processing (Fernandez et al., 2003; Eisner et al., 2004; Jacobs and D'Esposito, 2011). In addition to their known involvement in those brain functions, gonadal steroids are believed to influence the control of vocal communication. Evidence from birdsong research shows that estrogen helps maintain the plasticity of the song system to acquire new sensory models of song, and the lack of estrogen during the normal critical period of song learning impairs the development of adult song (Schlinger, 1997). In humans, fluctuations of gonadal steroids drive changes in voice quality such as roughness, breathiness, and asthenia (Meurer et al., 2009; Raj et al., 2010; Çelik et al., 2013) and may influence the timing of voice onset and offset (Wadnerkar et al., 2006). As compared to premenopausal women, postmenopausal women suffer from vocal deficits including lower voice fundamental frequency (F_0_), lower vocal intensity, and more voice instability, and their voice quality was improved with hormone replacement therapy (D'Haeseleer et al., 2009). Evidence suggests that the vocal folds contain specific estrogen and progesterone receptors in the vocalis muscle and lamina propria (Ferguson et al., 1987; Newman et al., 2000). Therefore, changes in these gonadal steroids may influence vocal motor behavior through the receptor-coupled effector mechanisms.

Both estradiol and progesterone can also influence auditory function. For example, exposing female midshipman fish to estradiol during the non-breeding season makes their auditory nerves more sensitive to the frequency of the male mating call (Sisneros et al., 2004). Acute inhibition of estradiol production in songbirds suppresses burst firing of auditory neurons and disrupts their ability to process and respond to song stimuli (Remage-Healey et al., 2010). In humans, auditory acuity, spontaneous otoacoustic emissions, and auditory processing assessed by auditory brainstem response (ABR) and auditory event-related potentials (ERPs) vary significantly as gonadal steroids change across the menstrual cycle (Serra et al., 2003; Walpurger et al., 2004; Al-Mana et al., 2010). For example, N1-P2 peak responses to pure tones were significantly reduced during the luteal phase as compared to the menstrual and follicular phase of the menstrual cycle in women, and they were negatively correlated with estradiol/progesterone levels (Walpurger et al., 2004). At the brainstem level, there was a significant increase in the wave V latency of ABR in the follicular phase and a decrease in the luteal phase (Al-Mana et al., 2010).

The control of speech motor behavior involves the integration of sensory information, particularly auditory information, into the vocal motor systems (Smotherman, 2007). Although gonadal steroids can influence both vocal motor behavior and central auditory processing, less is known about the interaction between gonadal steroids and auditory-motor integration for voice control in adult women. Auditory feedback provides information necessary to monitor and correct for errors for the guidance of speech learning during the critical phases of development, and for the maintenance of online speech production (Houde and Jordan, 1998; Jones and Munhall, 2005). When perceiving alterations of F_0_, intensity, or formant frequency (F1) in auditory feedback, people produce rapid compensatory vocal adjustment to stabilize their production of vocal sounds around the desired level (Burnett et al., 1998; Bauer et al., 2006; Purcell and Munhall, 2006). At the cortical level, N1 and P2 responses elicited by pitch-shifted voice auditory feedback can be modified by stimulus features (Liu et al., 2011a; Scheerer et al., 2013), attentional demands (Hu et al., 2015; Liu et al., 2015), and the nature of the vocalization task (Behroozmand et al., 2009, 2011). These two components are thought to reflect the detection and correction of feedback errors during the online monitoring of self-produced speech (Behroozmand et al., 2011; Guo et al., 2016). Also, a number of neuroimaging studies have revealed the brain regions involved in auditory-motor integration for voice control such as the superior temporal gyrus (STG), dorsal lateral prefrontal cortex (DLPFC), anterior cingulate cortex (ACC), premotor cortex (PMC), and inferior frontal gyrus (IFG) (Zarate and Zatorre, 2008; Parkinson et al., 2012; Chang et al., 2013; Behroozmand et al., 2015; Belyk et al., 2016). Despite the progress made in understanding the feedback-based processing of speech motor control, the biological influence of gonadal steroids on the auditory-vocal integration is largely unknown.

The lack of knowledge is striking, considering that gonadal steroids influence functional connectivity in the DLPFC and the ACC (Dreher et al., 2007); both brain regions are involved in auditory-vocal integration (Zarate and Zatorre, 2008). In addition, estradiol and progesterone act synergistically on the vocal musculo-mucosal complex (Abitbol et al., 1999). Previous studies have shown an important association between gonadal steroids and voice control. For example, female singers experience difficulties singing high notes, producing accurate tone, and with intricate phonation control just prior to menstruation (Lacina, 1968). Hormonal shifts in women experiencing menopause or Turner's syndrome have also been associated with significant problems with voice, speech, and hearing (Caras, 2013). Thus, increasing our understanding of the interplay between gonadal steroids and the auditory-vocal system has important implications for the gonadal steroid effects on prevalence and treatment of voice/speech disorders, in particular for women.

The menstrual cycle offers a unique opportunity to investigate how fluctuations of gonadal steroids modulate sensory processing to shape behavior in women. By using the frequency altered feedback (FAF) paradigm (Burnett et al., 1998), therefore, the present study sought to investigate the effects of gonadal steroids on auditory-motor integration for voice control in a counterbalanced, repeated-measures design during the menstrual, follicular, and luteal phases of the menstrual cycle. Participants were instructed to sustain a vowel phonation while exposed to unexpected pitch perturbations in their voice auditory feedback. We measured the magnitudes and latencies of vocal and ERP responses (N1 and P2) to pitch feedback perturbations and assessed plasma estradiol and progesterone levels across the menstrual cycle. We expected that fluctuations in gonadal steroids across the menstrual cycle would influence the auditory-motor processing of pitch feedback errors at the levels of behavior and cortex.

## Materials and methods

### Subjects

Nineteen female right-handed adults aged 19–31 years of old, who were students at Sun Yat-sen University of China, participated in the experiment. All participants were right-handed, native speakers of Mandarin Chinese. The following criteria led to inclusion in the study: nonuse of oral contraceptives, no hormone-replacement therapy, no history of lactation and pregnancy, regular monthly menstrual cycle, no diagnosed premenstrual syndrome, no intake of neuroactive substances (e.g., alcohol, caffeine, drugs, etc.), no prior history of neurological, psychiatric, or endocrinological illnesses, nonsmokers, no speech, language, or hearing disorders, and normal body weight (body mass index between 18.5 and 23.9 kg/m2). In all participants, pure-tone thresholds were ≤25 dB hearing level (HL) for octaves from 500 to 4000 Hz bilaterally.

Of 19 participants who eventually entered the study, 8 had to be excluded because they had anovulatory cycles during the sampling period (*N* = 6) or their electrophysiological data failed to reach the criteria of inclusion (*N* = 2) (see below). Therefore, the final study set comprised 11 participants with a mean age of 23 years [19–29 years; standard deviation (SD) = 3.9], reportedly regular menstrual cycle length (28–32 days), and a mean body mass index of 20.3 (18.5–22.4; SD = 2.2).

All participants provided written informed consent in compliance with a protocol approved by the Institution Review Board of The First Affiliated Hospital at Sun Yat-sen University of China in accordance with the Code of Ethics of the World Medical Association (Declaration of Helsinki).

### Hormone assessment

Three different phases of the menstrual cycle were investigated: menstrual phase (second to fourth day of bleeding), follicular phase (15–22 days before the onset of the new menstrual cycle), and luteal phase (3–9 days before the onset of the new menstrual cycle). During the menstrual phase, both the estradiol and progesterone levels were near baseline. The follicular phase is characterized by high estradiol and low progesterone levels, while both estradiol and progesterone levels are high during the luteal phase. Venous blood samples were collected 4 h prior to the experiment for determination of estradiol and progesterone levels in all three phases, leading to a total of 33 blood samples (3 phases × 11 subjects). Serum estradiol and progesterone levels were measured by chemiluminesecence immunoassay in the Immunology Laboratory of The First Affiliated Hospital at Sun Yat-sen University of China. For the estradiol, the assay sensitivity was 5.00 pg/ml, and the inter- and intra-assay coefficients of variation (CV) were 3.6 and 2.2%, respectively. For the progesterone, the assay sensitivity was 0.03 ng/ml, and the inter- and intra-assay CVs were 4.6 and 2.3%, respectively. A menstrual cycle was classified as anovulatory if progesterone levels did not rise above 5 ng/ml (Herzog et al., 1997).

### Apparatus

Throughout the experiment, all participants sat in a sound-treated booth. Prior to the data recording, the experimental system was acoustically calibrated to ensure that participants heard the voice feedback with a gain of 10 dB sound pressure level (SPL) relative to the intensity of their vocal output. This gain was used to partially mask air-born and bone-conducted voice feedback (Behroozmand et al., 2009). Voice signals were recorded through a dynamic microphone (model DM2200, Takstar Inc.) and amplified with a MOTU Ultralite Mk3 Firewire audio interface. The amplified signals were then pitch-shifted by an Eventide Eclipse Harmonizer controlled by a custom-developed program (Max/MSP, v.5.0, Cycling 74). Direction, magnitude, and duration of the pitch perturbation and the inter-stimulus interval (ISI) were controlled by this program. Transistor-transistor logic (TTL) control pulses were generated to mark the onset and offset of the pitch perturbations. Finally, the pitch-shifted voices were amplified with an ICON NeoAmp headphone amplifier and fed back to participants through insert earphones (ER1-14A, Etymotic Research Inc.). The original voice, feedback and TTL control pulses were sampled at 10 kHz by a PowerLab A/D converter (model ML880, AD Instruments), and recorded using LabChart software (v.7.0 by AD Instruments).

To signal the onset of the pitch perturbations, we used a DIN synch cable to send the TTL control pulses to the electroencephalograph (EEG) recording system (Electrical Geodesics Inc., Eugene, OR). The EEG signals were recorded using a 64-electrode Geodesic Sensor Net, amplified by a Net Amps 300 amplifier, and saved onto a Mac Pro computer using NetStation software (v. 4.5, Electrical Geodesics Inc., Eugene, OR) with a sampling frequency of 1 kHz. During the online recording, the EEG signals across all channels were referenced to the vertex (Cz). Individual sensors were carefully adjusted to ensure that their impedance levels were <50kΩ throughout the EEG recording (Ferree et al., 2001).

### Experimental design

During each phase, participants completed the FAF-based vocal production experiment. We counterbalanced the menstrual cycle phases and randomly assigned subjects to perform the vocal tasks in a different combination of the menstrual cycle phase orders. Specifically, three of 11 participants included were tested first in their menstrual phase; 4 were tested first in their follicular phase; 4 were tested in their luteal phase. In the FAF-based vocal production experiment, participants were instructed to vocalize the vowel sound /u/ for approximately 5–6 s at their conversational pitch and loudness level. During each vocalization, participants heard their voice pitch-shifted downwards five times. The first stimulus occurred 500–1000 ms after the vocal onset, and the succeeding stimuli were presented with an ISI of 700–900 ms. Participants were asked to take a break of 2–3 s prior to initiating the next vocalization. Production of 20 consecutive vocalizations constituted one experimental block. A total of 100 trials were thus generated per block, within which the magnitude was held constant at −50 or −200 cents (100 cents = one semitone) and the duration of each perturbation was fixed at 200 ms. The magnitude of pitch perturbation was manipulated because previous research has shown its effect on the resultant behavioral and cortical responses (Behroozmand et al., 2009; Scheerer et al., 2013). The order of the two blocks was counterbalanced across all participants.

### Vocal responses measurement

Digitized voice and feedback signals were analyzed offline using event-related averaging techniques (Liu et al., 2011b). First, pulses for each glottal cycle in the voice signals were generated using Praat (Boersma, 2001), and were converted to voice F_0_ contours in Hz in IGOR PRO (v.6.0, Wavemetrics Inc.). Secondly, the F_0_ values in Hz were converted to the cent scale using the formula: cents = 100 × (12 × log_2_(F_0_/reference)), where 195.997 Hz (G4) served as the arbitrary reference. Voice F_0_ contours were then segmented starting 200 ms before and ending 700 ms after the onset of the pitch perturbation. A visual inspection was performed on all individual trials to ensure that trials that were the result of vocal interruption or signal processing errors were excluded from further analyses. Finally, artifact-free trials were normalized by subtracting the mean F_0_ values in the baseline period (−200 to 0 ms) from the F_0_ values after the onset of the pitch perturbation and averaged to generate an overall response for each condition. An acceptable response was defined as one in which the contours had to exceed a value of two SDs of the pre-stimulus mean beginning at least 60 ms after the stimulus onset and lasting at least 50 ms. Response latency in milliseconds was determined as the time at which the response exceeded 2 SDs above or below the pre-stimulus mean following the perturbation onset. Response magnitude in cents was measured as the difference between the pre-stimulus mean and the peak value of the voice F_0_ contour following the response onset.

### EEG data analyses

The EEG signals were sent to NetStation software for offline analyses. All channels were re-referenced to the average of electrodes on each mastoid and band-passed filtered at 1–20 Hz. The continuous EEG data was segmented into epochs with a window of 200 ms before and 500 ms after the onset of the pitch perturbation. Segmented trials were scanned for artifact contamination such as excessive muscular activity, eye blinks, and eye movement using the Artifact Detection toolbox in NetStation. Additional visual inspection was performed to ensure that artifacts were appropriately rejected. Individual electrodes were rejected if they contained artifacts more than 20% of the segments, and the file was excluded for further analyses if it contained more than 10 bad channels. Two participants mentioned above were excluded from further analyses due to high rejection rates (over 50% of trials). Overall, 82% of trials were retained across all participants. Finally, artifact-free trials were averaged and baseline-corrected for each condition to generate an overall response. The amplitudes and latencies of N1 and P2 components were measured from 10 electrodes (FC1, FC2, FCz, FC3, FC4, C1, C2, Cz, C3, and C4) as the negative and positive peaks in the time windows of 80–180 m and 160–280 ms after the onset of pitch perturbation and submitted to statistical analyses. These electrodes were chosen because cortical responses to pitch perturbations in voice auditory feedback are primarily pronounced in the N1 and P2 components recorded from the frontal-central electrodes (Hawco et al., 2009; Chen et al., 2012).

### Statistical analyses

Values of vocal and ERP responses to pitch perturbations across conditions were subjected to repeated-measures analysis of variance (RM-ANOVAs) in SPSS (v.16.0). The magnitudes and latencies of vocal responses were subjected to two-way RM-ANOVAs, in which pitch shift (−50 and −200 cents) and menstrual cycle phase (menstrual, follicular, and luteal phase) were regarded as within-subject factors. Three-way RM-ANOVAs were used to analyze the amplitudes and the latencies of the N1 and P2 responses, including within-subjects factors of pitch shift, menstrual cycle phase, and electrode site. Subsidiary RM-ANOVAs were conducted if higher-order interactions reached significance. Probability values were corrected for multiple degrees of freedom using Greenhouse-Geisser if the assumption of sphericity was violated. As well, estradiol and progesterone data were subjected to one-way RM-ANOVAs with the within-subject factor of menstrual cycle phase. In addition, linear correlations using Pearson correlation coefficients were performed to examine the relationship between estradiol/progesterone levels and vocal/ERP responses to pitch perturbations.

## Results

### Hormonal data

As expected, there was a significant main effect of menstrual cycle phase for estradiol [*F*_(2, 20)_ = 19.054, *p* < 0.001], in which there was a significant decrease in estradiol levels during the menstrual phase (22.6 ± 2.1 pg/ml) (mean ± standard error, same as below) as compared to the follicular phase (109.9 ± 18.2 pg/ml) (*p* = 0.001) and the luteal phase (98.7 ± 9.8 pg/ml) (*p* < 0.001). Similarly, the main effect of menstrual cycle phase for progesterone also reached significance [*F*_(2, 20)_ = 42.674, *p* < 0.001], in which the luteal phase (9.0 ± 1.4 ng/ml) was associated with higher progesterone levels than both the menstrual (0.2 ± 0.02 ng/ml) (*p* < 0.001) and follicular phases (0.3 ± 0.06 ng/ml) (*p* < 0.001).

### Vocal responses

**Figure 1:**
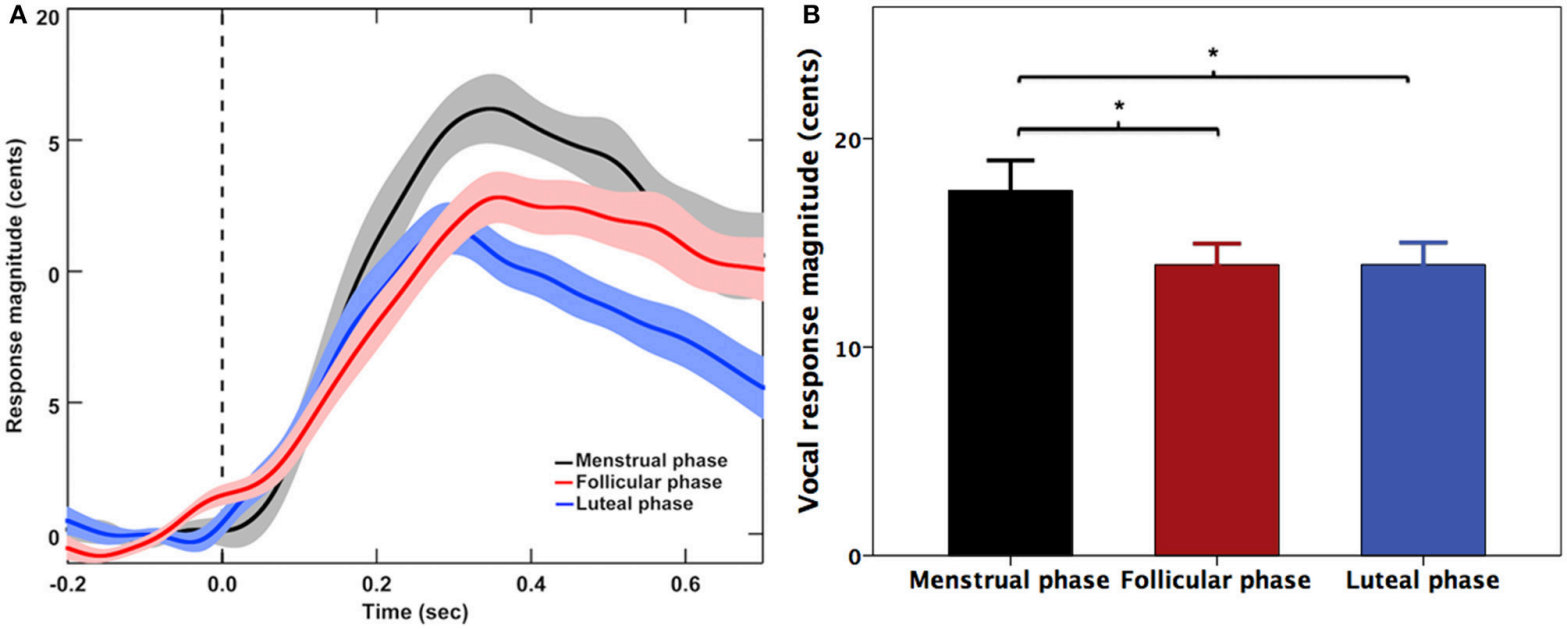
**(A)** Grand-averaged voice F_0_ contours and **(B)** T-bar graphs (means and standard errors) of the magnitude of vocal responses to pitch perturbations across three menstrual cycle phases. The solid lines and bars in black, red, and blue represent the vocal responses during the menstrual, follicular, and luteal phases. The asterisks indicate significantly larger response magnitudes during the menstrual phase when compared with the follicular phase and luteal phase.

Figure 1 shows the grand-averaged voice F_0_ contours (A) and the T-bar plots (B) of the magnitudes of vocal responses to pitch-shifted auditory feedback. One two-way RM-ANOVA conducted on the magnitudes of vocal responses revealed a significant main effect of menstrual cycle phase [*F*_(2, 20)_ = 5.888, *p* = 0.023], indicating a significant increase of response magnitude during the menstrual phase when compared with the follicular phase (*p* = 0.040) and luteal phase (*p* = 0.025) (See Figure 1B). The main effect of pitch shift [*F*_(1, 10)_ = 0.408, *p* = 0.537] and the interaction between menstrual cycle phase and pitch shift [*F*_(2, 20)_ = 0.531, *p* = 0.549] did not reach significance. For the latencies of vocal responses, there were no significant main effects of menstrual cycle phase [*F*_(2, 20)_ = 0.420, *p* = 0.581], pitch shift [*F*_(1, 10)_ = 2.873, *p* = 0.121] and their interactions [*F*_(2, 20)_ = 1.397, *p* = 0.271].

### ERP findings

**Figure 2:**
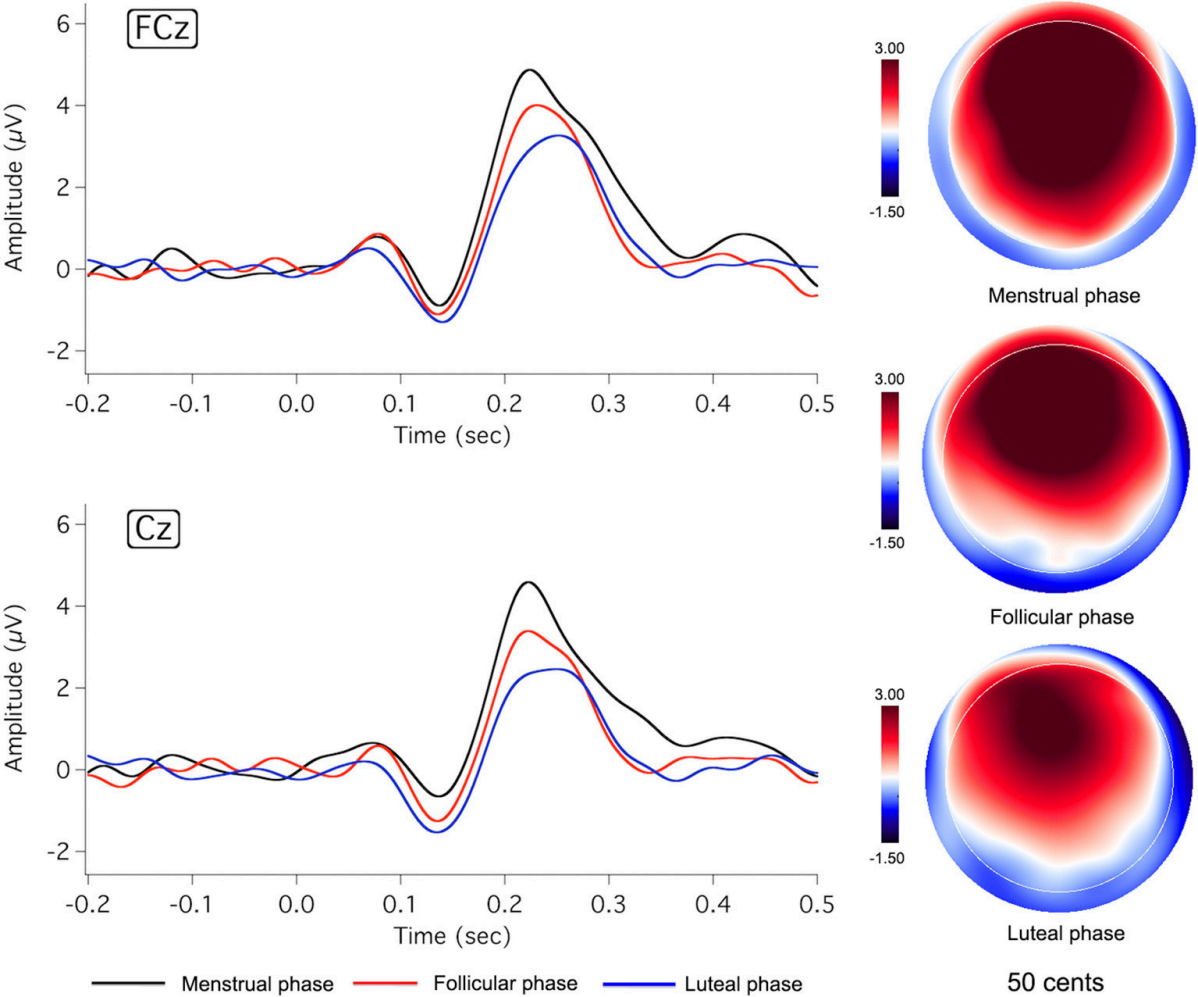
**Grand-averaged ERP waveforms (left)** in responses to pitch-shift stimuli of −50 cents during the menstrual (black lines), follicular (red lines), and luteal phases (blue lines), and topographic distributions of P2 amplitudes (right) during the menstrual (**top:** latency = 233 ms), follicular (**middle:** latency = 227 ms), and luteal phases (**bottom:** latency = 242 ms).

**Figure 3:**
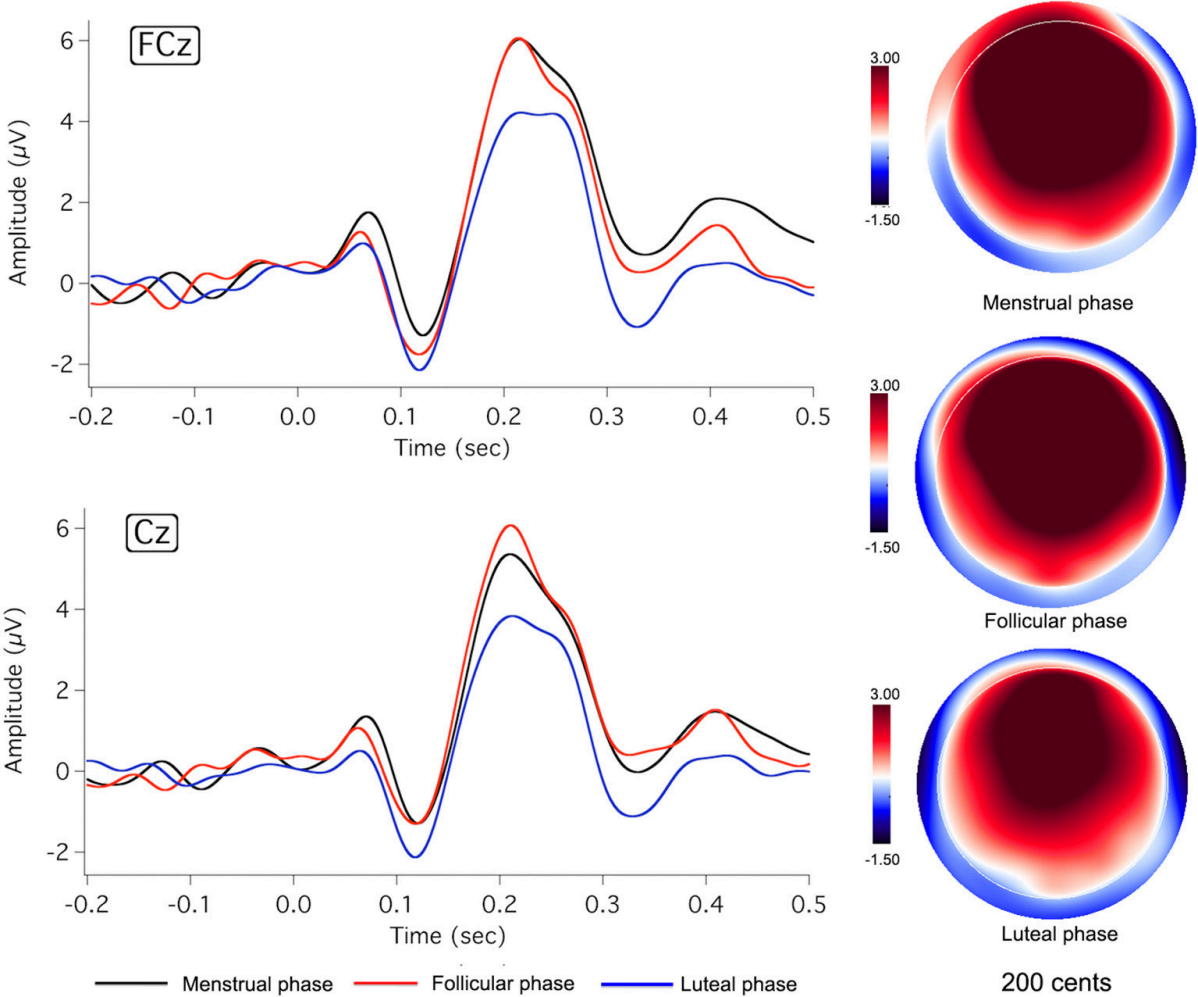
**Grand-averaged ERP waveforms (left)** in response to pitch-shift stimuli of −200 cents during the menstrual (black lines), follicular (red lines), and luteal phases (blue lines), and topographic distributions of P2 amplitudes (right) during the menstrual (**top:** latency = 218 ms), follicular (middle: latency = 223 ms), and luteal phases (**bottom:** latency = 221 ms).

Figures 2, 3 show the grand-averaged ERP waveforms (left column) and topographical distributions of P2 responses (right column) as a function of menstrual cycle phase for the −50 and −200 cents conditions, respectively. As can be seen, change in cortical responses to pitch perturbations across the three menstrual cycle phases was primarily reflected in the P2 component. The luteal phase (blue lines) was associated with smaller P2 amplitudes than the menstrual (black lines) and follicular phases (red lines). This change can also be seen in the topographical distributions of P2 responses in Figures 2, 3. In contrast, N1 responses appeared to remain constant across the menstrual cycle phase.

**Figure 4:**
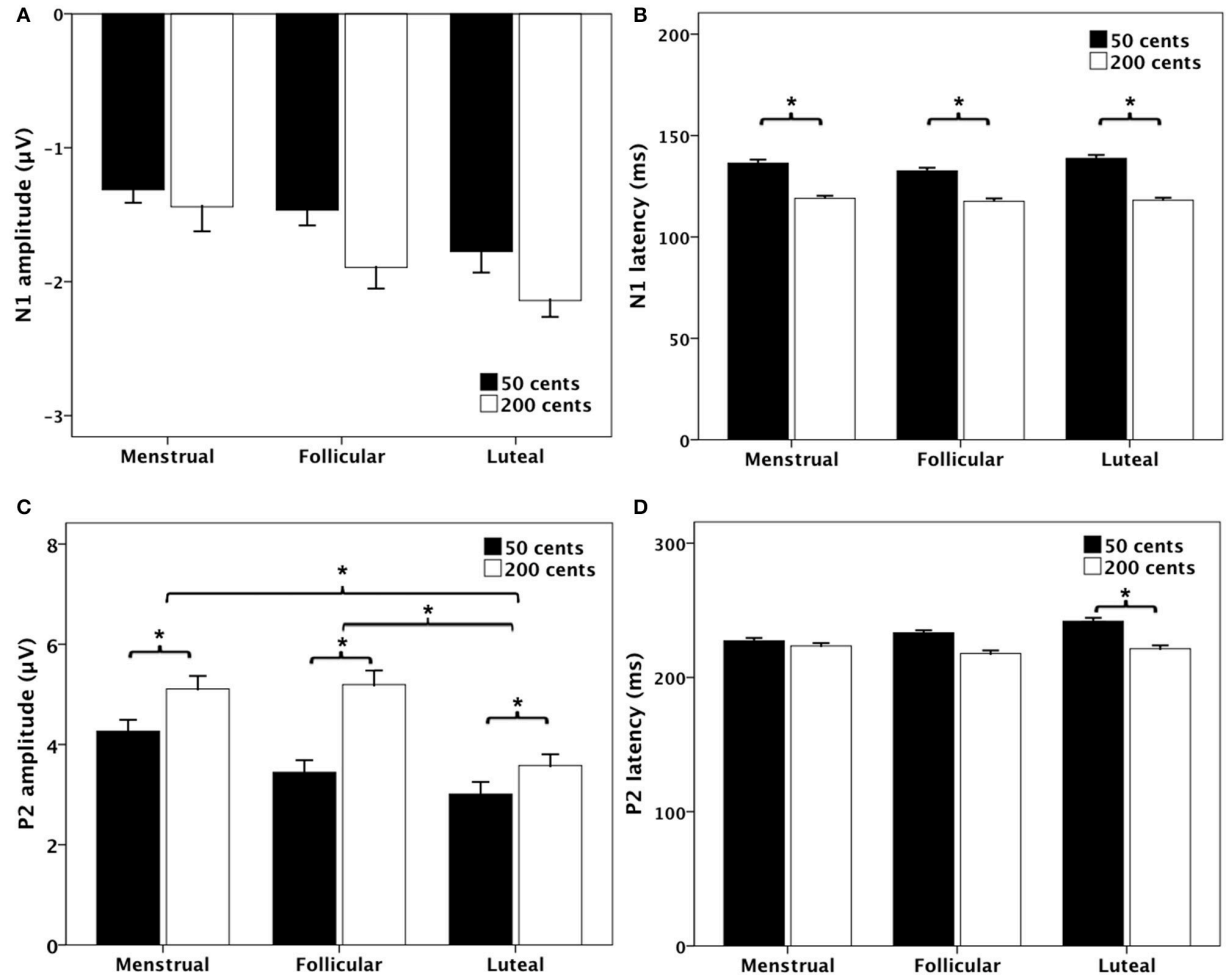
**Bar graphs (means and standard errors) of the magnitudes and latencies of N1 (A,B)** and P2 **(C,D)** components as a function of stimulus and phase. The black and the blank bars denote the responses to −50 and −200 cents, respectively. The asterisks indicate significant differences between conditions.

Figure 4 shows the T-bar graphs of the N1 and P2 responses as a function of menstrual cycle phase for the −50 (black bars) and −200 cents (blank bars) conditions. A three-way RM-ANOVA conducted on the N1 amplitudes revealed a significant main effect of electrode site [*F*_(9, 90)_ = 3.363, *p* = 0.033], but N1 amplitudes did not differ as a function of menstrual cycle phase [*F*_(2, 20)_ = 1.718, *p* = 0.205] or pitch shift [*F*_(1, 10)_ = 1.049, *p* = 0.330] (see Figure 4A). As for the N1 latencies, the −50 cents condition elicited significantly longer N1 latencies than the −200 cents condition [*F*_(1, 10)_ = 21.561, *p* = 0.001] (see Figure 4B). The main effects of menstrual cycle phase [*F*_(2, 20)_ = 0.564, *p* = 0.578] and electrode site [*F*_(9, 90)_ = 2.034, *p* = 0.137], however, did not reach significance. Neither did the interactions between any of these variables (*p* > 0.05).

A three-way RM-ANOVA conducted on the P2 amplitudes revealed significant main effects of menstrual cycle phase [*F*_(2, 20)_ = 12.479, *p* < 0.001], pitch shift [*F*_(1, 10)_ = 7.916, *p* = 0.018], and electrode site [*F*_(9, 90)_ = 20.648, *p* < 0.001]. *Post-hoc* Bonferroni comparison revealed that P2 amplitudes were significantly smaller during the luteal phase than that during the menstrual phase (*p* = 0.007) and follicular phase (*p* < 0.001), while the difference between the menstrual and follicular phases did not reach significance (*p* = 0.850) (see Figure 4C). In addition, the −50 cents condition elicited significantly smaller P2 amplitudes than −200 cents condition (*p* = 0.018). There were no significant interactions between any of these variables (*p* > 0.05).

Regarding the P2 latencies, main effects of menstrual cycle phase [*F*_(2, 20)_ = 0.652, *p* = 0.476], pitch shift [*F*_(1, 10)_ = 3.209, *p* = 0.103], and electrode site [*F*_(9, 90)_ = 1.080, *p* = 0.366] did not reach significance. A significant interaction, however, was found between menstrual cycle phase and pitch shift [*F*_(2, 20)_ = 4.912, *p* = 0.018]. Follow-up two-way RM-ANOVAs revealed that the −50 cents condition elicited significantly longer P2 latencies than the −200 cents condition [*F*_(1, 10)_ = 6.549, *p* = 0.028] during the luteal phase (see Figure 4D). The main effect of pitch shift on the P2 latencies, however, did not reach significance during the menstrual phase [*F*_(1, 10)_ = 0.177, *p* = 0.683] or follicular phase [*F*_(1, 10)_ = 4.771, *p* = 0.054].

**Figure 5:**
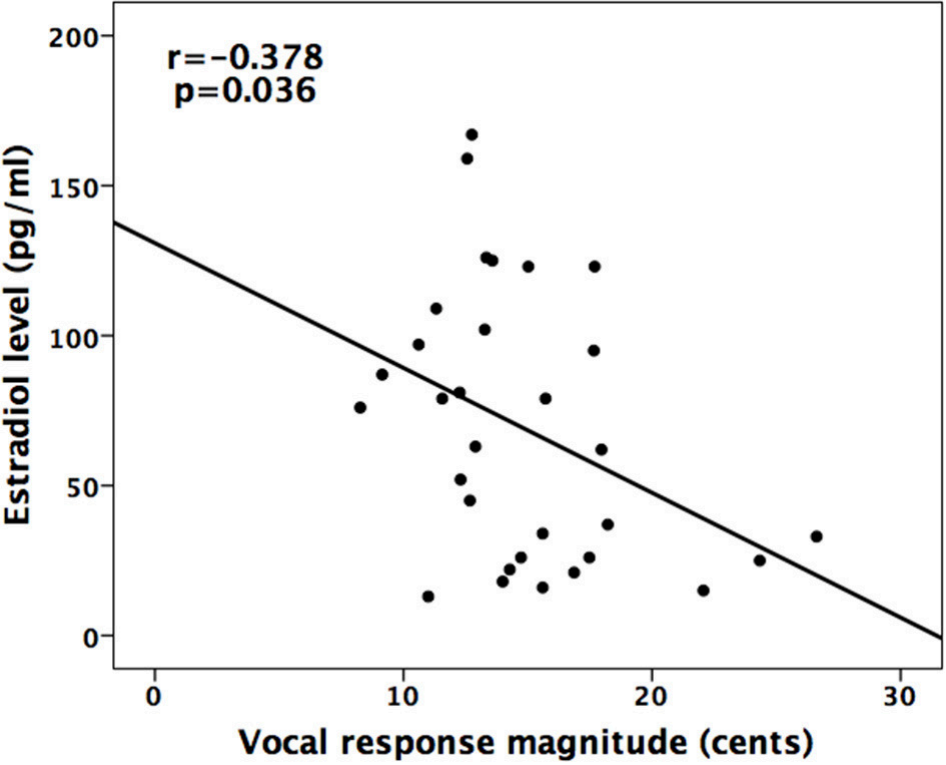
**Scatter plot of the magnitudes of vocal responses to pitch perturbations as a function of estradiol level**. There was a negative correlation between estradiol level and vocal response magnitude (*R*^2^ = 0.143, *p* = 0.036).

In addition, linear correlation analyses were performed to examine the relationship between the magnitudes of vocal and ERP (P2) responses and estradiol/progesterone concentrations across the menstrual cycle phase. The results revealed a significant negative correlation between the mean magnitudes of vocal responses and estradiol levels (*R*^2^ = 0.143, *p* = 0.036), indicating that higher estradiol levels are associated smaller magnitudes of vocal responses (see Figure 5). The mean magnitudes of vocal responses, however, were not significantly correlated with progesterone levels (*R*^2^ = 0.016, *p* = 0.482). In addition, the mean amplitudes of P2 responses were not correlated with estradiol (*R*^2^ = 0.021, *p* = 0.425) and progesterone levels (*R*^2^ = 0.006, *p* = 0.659).

## Discussion

The present study investigated the influence of gonadal steroids on auditory-motor integration for voice control across the menstrual cycle in young adult women. The behavioral findings showed a significant increase in the magnitudes of vocal responses during the menstrual phase when estradiol levels were low, and that there was a significant negative correlation between the magnitudes of vocal responses and estradiol levels. At the cortical level, P2 amplitudes were significantly smaller during the luteal phase when progesterone levels were high when compared to the menstrual and follicular phases. These findings provide evidence for the modulation of neurobehavioral responses to pitch-shifted voice auditory feedback by menstrual cycle phase, suggesting that gonadal steroids, particularly estradiol, may have a modulatory effect on the auditory-motor processing of feedback errors during vocal pitch regulation.

Previous behavioral studies have demonstrated that changes in gonadal steroids can influence the perceptual features (e.g., roughness, breathiness, asthenia, etc.) of the voice across the menstrual cycle (Meurer et al., 2009; Raj et al., 2010; Çelik et al., 2013). For example, using GRBAS scale of perceptual evaluation, Çelik et al. (2013) reported that voice quality was at the highest level during the mid-menstrual period when estrogen and progesterone levels were high and showed a significant decrease during the premenstrual period when those hormones levels were low. Findings from the present study further show that auditory-motor integration for voice control can be modulated as a function of menstrual cycle phase. Our behavioral findings revealed larger vocal responses to pitch-shifted auditory feedback during the menstrual phase when estradiol levels were low and a significant negative correlation between them. This finding is in line with another study showing shorter VOT values for the voiced plosives and longer VOT values for the voiceless plosives in the phase with high estradiol and progesterone levels (Wadnerkar et al., 2006). On the other hand, the present study showed a significant decrease of P2 amplitudes during the luteal phase when progesterone levels were high. This finding is complimentary to previous studies that showed decreased cortical responses (N1-P2 peak amplitude) or shorter subcortical responses (ABR) to pure tones during the luteal phase when the progesterone levels were high in women (Serra et al., 2003; Walpurger et al., 2004; Al-Mana et al., 2010).

Our behavioral results revealed larger vocal responses to pitch perturbations during the menstrual phase relative to the follicular and luteal phases, while higher estradiol levels were associated with the menstrual phase than the follicular and luteal phase. Furthermore, Pearson correlation analyses revealed a significant negative correlation between the magnitudes of vocal responses and estradiol levels, suggesting a possible interaction between vocal compensation for pitch feedback errors and estradiol concentration. The effects of estradiol on the vocal motor behavior have been documented in animal studies. For example, the duration of fictive vocalization produced by the midshipman fish is rapidly responsive to steroid hormones including androgens and estrogens (Remage-Healey and Bass, 2004), and behavioral selectivity of conspecific vocalizations can be enhanced through modulating the synthesis of estradiol in seasonally-breeding songbirds (Caras, 2013). Although direct evidence of estradiol effect on vocal motor output in humans is scarce, it has been reported that specific estradiol receptors have been identified in the normal human larynx (Ferguson et al., 1987; Newman et al., 2000). By activating receptor-coupled effector mechanisms through binding to those specific receptors in the larynx, estradiol may be capable of directly influencing the female laryngeal muscles such as the cricohthyroid and thyroarytenoid muscles (Liu et al., 2011c) to modulate the vocal compensation for pitch-shifted auditory feedback. On the other hand, the neural substrates involved in the auditory-vocal integration include the STG, PMC, IFG, DLPFC, and ACC (Zarate and Zatorre, 2008; Behroozmand et al., 2015). Estradiol passes the blood-brain-barrier and binds to receptors in various parts of the brain, including the frontal and middle temporal lobes (Goldstein et al., 2001; Ostlund et al., 2003). Thus, estradiol may also influence vocal motor function through the modulation of these receptors in the neural substrates involved in the feedback-based voice control. Taken together, changes in vocal responses to pitch-shifted auditory feedback may be caused by estradiol through the specific receptors that exist in the larynx and the neural substrates involved in the auditory-vocal integration.

The present study also revealed a significant decrease in the amplitudes of P2 responses during the luteal phase when progesterone levels were high. This is consistent with previous findings that showed the inhibitory effect of progesterone on the central auditory processing. For example, the N1-P2 peak amplitude to neutral auditory stimuli was significantly decreased during the luteal phase and negatively correlated to the progesterone concentration (Walpurger et al., 2004). Postmenopausal females treated with estradiol alone had better performance of speech perception in noise than did those treated with both estradiol and progesterone (Guimaraes et al., 2006). Accumulating evidence has shown that P2 component reflects not only the central auditory processing (e.g., error detection) but also the feedback-based motor processing (e.g., error correction) in the online monitoring of vocal production. For example, when compensating for pitch perturbations in voice auditory feedback, individuals with Parkinson's disease produced significantly larger P2 responses than healthy controls due to enhanced activity in the brain regions including the STG, PMC, and IFG (Huang et al., 2016). Also, P2 responses were found to be significantly correlated with regional homogeneity of those brain regions in the resting-state (Guo et al., 2016). Thus, decreased P2 responses during the luteal phase rise observed in the present study are indicative of the influence of menstrual cycle phase on the cortical processing of vocal pitch regulation.

Although P2 responses were not significantly correlated with progesterone levels, we speculate that the observable decreased P2 responses with the luteal phase rise in progesterone might be partly related to the inhibitory effect of progesterone and its metabolites on the central auditory system through their interaction with the steroid binding sites on γ-aminobutyric acid-A (GABA-A) receptors (Follesa et al., 2001). Allopregnanolone, a progesterone metabolite, has been observed to positively modulate GABA-A receptor-evoked responses in cortical neurons (Stell et al., 2003), and its brain level mirrors changes in progesterone (Wang et al., 1996). 5-hydroxytryptamine (5-HT) is thought to be involved in the central auditory processing (Juckel et al., 1999), and high levels of allopregnanolone can enhance GABA-A receptor-mediated responses and inhibit 5-HT neuronal activity, leading to a decrease in neuronal excitability. The greatest GABA-A receptor inhibition of 5-HT release was found at times when progesterone and hence allopregnanolone, levels were highest (Felton and Auerbach, 2004), and allopregenanolone significantly potentiated the inhibitory responses of 5-HT neurons to GABA-A receptor activation (Kaura et al., 2007). Accordingly, a potential mechanism underlying the association between the decreased cortical responses to feedback perturbations and the luteal phase rise in progesterone in women may include an allopregnanolone-mediated potentiation of GABAergic inhibition of serotonergic transmission.

In addition to the individual actions of estradiol and progesterone, we cannot rule out the possibility that fluctuations of both estradiol and progesterone may make contributions to the modulation of neurobehavioral responses to pitch-shifted voice auditory feedback across the menstrual cycle. For example, participants with high estrogen levels produced smaller mismatch negativity (MMN) to neutral auditory stimuli than did participants with low estrogen levels (Schirmer et al., 2008), and women with Turner's syndrome who are estrogen deficient have higher rates of hearing decline and abnormal ABR relative to general population (Caras, 2013), suggesting the influence of estrogen on the central auditory processing (Pinaud and Tremere, 2012). On the other hand, the motor evoked potential, or the amplitude of the muscle response, has been shown to be significantly reduced during the luteal phase relative to the follicular phase in women, indicating an inhibitory effect of progesterone on the motor cortex (Smith et al., 2002, 2003). Moreover, some studies have reported a coadjuvant effect of estrogen and progesterone. Estradiol has been shown to induce nuclear progesterone receptors in some brain areas, and estrogen treatment is required for progesterone-evoked dopamine release or progesterone-related sexual behaviors (Dluzen and Ramirez, 1990; Moffatt et al., 1998). Given the complex interplay of the central nervous system with the reproductive system and immune system, further studies with a larger sample size should be conducted to explore this sensory-neuroendocrine interaction in speech motor control.

Overall, the present study probed the interaction between gonadal steroids and auditory-motor integration for voice control across the menstrual cycle. The results showed the modulation of vocal and cortical responses in compensating for pitch errors in voice auditory feedback across the menstrual cycle phase and revealed a significant negative correlation between the magnitudes of vocal responses and estradiol levels. Despite the poor understanding of the biological mechanisms underlying the regulation of auditory-vocal integration, we believe that these findings provide neurobehavioral evidence for the impact of menstrual cycle on the auditory-motor integration in humans. Our results suggest that caution must be exercised in interpreting the impact of menstrual variation on behavioral performance in women of reproductive age.

## Author contributions

XZ, YN, XC, and HL designed the experiment; XZ, YN, WL, and ZZ performed the experiment and analyzed the data; XZ, YN, WL, PL, XC, and HL interpreted the results and wrote the manuscript; all authors read and approved the final manuscript.

### Conflict of interest statement

The authors declare that the research was conducted in the absence of any commercial or financial relationships that could be construed as a potential conflict of interest.
